# Differentiating between Underwater Construction Noise of Monopile and Jacket Foundations for Offshore Windmills: A Case Study from the Belgian Part of the North Sea

**DOI:** 10.1155/2013/897624

**Published:** 2013-03-18

**Authors:** Alain Michel Jules Norro, Bob Rumes, Steven Johan Degraer

**Affiliations:** Royal Belgian Institute of Natural Sciences, Management Unit of the North Sea Mathematical Models, Gulledelle 100, 1200 Brussels, Belgium

## Abstract

Steel monopiles, jackets requiring four steel pinpiles, and gravity-based foundations were applied in offshore wind farms in the Belgian part of the North Sea. This paper compares the underwater noise generated during the piling activities of steel monopiles at the Belwind wind farm (Blighbank) with that of jacket pinpiles at the C-Power project (Thorntonbank). Underwater noise was measured at various distances from the pile driving location. The underwater noise was quantified by its zero to peak sound pressure level (*L*_*z*−*p*_), unweighted sound exposure level (SEL), cumulative SEL, and 1/3 octave spectra. No significant differences in *L*_*z*−*p*_ could be demonstrated (monopile *L*_*z*−*p*_: 179–194 dB re 1 **μ**Pa, jacket *L*_*z*−*p*_: 172–189 dB re 1 **μ**Pa). SEL showed no statistical difference between monopile and jacket and varied between 145 and 168 dB re 1 **μ**Pa^2^s. Furthermore, near identical spectra were measured for both types of piling. Piling of the jacket pinpiles took, however, about 2.5 times the time of the monopile. When standardised to megawatt installed per foundation both types of piling scored near equally. As an illustration, the radius of major behavioural disturbance (*L*_*p*−*p*_ = 155 dB re 1 **μ**Pa) in the harbour porpoise *Phocoena phocoena* was estimated by a model at 16 km for monopiles and at 8 km for jacket.

## 1. Introduction

The European Marine Strategy Framework Directive obliges every member state to achieve or maintain good environmental status, under which also the introduction of energy including underwater noise is considered a main concern [1]. An indicator for impulsive sound and a second indicator concerning the evolution of background noise are introduced. Clarification and details can be found in [2].

One of the major concerns in excessive underwater noise emissions is linked to offshore wind farms, as this industry is relatively new to the marine environment [3], is developing fast, and is highly diverse in technology used [4]. As such, at present, major attention is paid to the underwater noise generated during the construction, operation, and (future) dismantlement of offshore wind farms [3]. Here, four different phases should be distinguished in relation to the life cycle of an offshore wind farm: (1) the before implantation phase-reference situation, (2) the construction phase, (3) the operational phase, and (4) the dismantlement phase [5].

For the Belgian part of the North Sea (BPNS), the underwater noise emissions were documented for the first three phases, with reference sound pressure levels (SPL) of about 100 dB re 1 *μ*Pa at the Thorntonbank and Blighbank [6, 7]. So far, seven wind farms are planned for the BPNS, of which four have been granted both a domain concession and environmental permit. Two wind farms have actually been constructed. The first six windmills (C-Power project, phase 1; Thorntonbank) were built on concrete gravity based foundation (GBF), while in a second and third phase jacket foundations, involving the piling of four pinpiles per jacket, were used. In a second wind farm (Belwind project, Blighbank) only monopile foundations were applied. During the operational phase finally [8], a 20 dB re 1 *μ*Pa increase in mean SPL emitted in case of a steel monopile foundation (totalling 120 dB re 1 *μ*Pa at 100 Hz) was measured, while hardly any increase in underwater noise was observed in case of GBFs. 

This paper focuses on the differences in underwater noise emissions by two different types of piling, that is, piling of large monopiles (further called: monopiling) and the piling of the jacket foundation pinpiles (further called: jacket piling). In addition to zero to peak level (*L*_*z*−*p*_), the best measures for comparing noise from pile driving also include sound exposure level (SEL), as the latter is better related to the energy emitted by the piling. Comparison of both piling activities therefore focused on both *L*_*z*−*p*_ and SEL. We finally also compared their noise spectra and attenuation functions. As an illustration and for the harbour porpoise that is the only marine mammal present in high density in Belgian waters, some computations related to the impact of underwater noise levels are proposed.

## 2. Materials and Methods

Analysis focused on the quantification of the discontinuous impulsive pile driving-generated underwater noise. Underwater noise was measured at various distances (250–14000 m) from the pile driving location during the installation of steel monopiles and jackets at the Blighbank and Thorntonbank site, respectively. Zero to peak sound pressure level (*L*_*z*−*p*_), unweighted sound exposure level (SEL), cumulative SEL, and 1/3 octave spectra were computed in order to quantify the underwater noise emitted during the construction phase. 

### 2.1. Measurement Methodology

Measurements of wind farm construction noise were performed from a drifting rigid hull inflatable boat (RHIB) in the vicinity of the piling site [7]. To avoid interaction with the hydrophone, the engine, radar, and echosounder were turned off. The geographic position and time of measurement were recorded with a handheld GPS GARMIN GPSMap60 at a frequency of one position every 5 seconds. The clock of the recorder was synchronised beforehand with the GPS-time (UTC). At the start and the end of each measurement a reference signal was recorded. 

Several recordings of few minutes each (1 to 5 min.) were performed at different locations on September 26 2009 (monopile A02) and January 15 2010 (monopile B10) at the Blighbank and on the May 11 (jacket CG3) and the of July 12 2011 (jacket CB6) at the Thorntonbank site (Table 1). Weather conditions encountered during fieldwork featured a wind force of 1–3 BF and a sea state of 1 to 2.

### 2.2. Acoustic Measurement Equipment

For every measurement, a Brüel & Kjær hydrophone (type 8104) was deployed at a depth of 10 m. A Brüel & Kjær amplifier (Nexus type 2692-0S4) was connected between the hydrophone and the recorder in order to allow for an amplification and filtration of the signal. A reference signal was used together with the output sensitivity of the Nexus to calibrate the amplitude of the recorded signal. The signal was recorded using an audio MARANTZ Solid State Recorder (type PMD671). It was operated with the highest possible sampling rate of 44100 Hz. The signal was recorded in WAVE format (.wav) on Compact Flash cards of 2 GB (Sandisk Ultra II). Batteries powered all equipment. 

### 2.3. Response Variables

It is very common in underwater acoustics to use values expressed in a logarithmic scale (decibels). In order to characterize extreme level values of a transient signal like the one associated with pile driving the peak sound pressure level is often used. This terminology is not totally unambiguous and we prefer to use *L*_*z*−*p*_ that is defined by [9] as

(1) $$\begin{array}{c} L_{z-p}=10\log_{10}\frac{p_{z-p}^{2}}{p_{\text{ref}}^{2}}\;\text{in}\;\;\text{dB}\;\;\text{re}\;\;1\;\mu \text{Pa}. \end{array}$$

For impulsive sound, however, the unweighted SEL better characterises the energy produced by a given stroke, extracted from a complete piling event. SEL is computed as defined by [9]. The SEL is the level of a continuous sound during the integration period and having the same sound energy as the impulse:

(2) SEL=10log⁡⁡(1T  ∫T1T2p(t)2p02dt)=10log⁡⁡EEref in  dB  re  1 μPa2s,

where *T* is 1 second, *T*1 and *T*2 are, respectively, the start and the end of the integration time window (the complete stroke being included in this window), *p*(*t*) is the sound pressure signal, and *p*_0_ is the reference sound pressure of 1 *μ*Pa. When more than one noise pulse is generated as is the case for pile driving, it is possible to compute a cumulative sound exposure level. For a series of strokes, the cumulative SEL is computed following the definition given by [10], advising not to rely only on cumulative SEL but also to include the total number of blows and the frequency of piling. Measurements made at various distances were normalized to a reference distance of 750 m using the equation [11, 12]:

(3) Lnorm⁡=Lmeasured+15log⁡10⁡(distance750).  

This normalization has been used in this study in order to allow for an appropriate comparison of noise characteristics collected at various distances from the source using a normalized transmission loss [11, 12] permitting comparison with other sites.

The third octave band spectrum of the underwater sound pressure level was computed according to the norm IEC1260. All these computations were made using dedicated routines developed using the MATLAB environment. 

A Kruskal-Wallis test, followed by Dunn's *post hoc* multiple comparison tests, was used to identify statistically significant differences in the underwater noise emitted by the different foundation types. More specifically, Dunn's *post hoc* test as applied by Statistica 10 compares the difference in the sum of ranks between two columns with the expected average difference (based on the number of groups and their size). For each pair of columns, Prism reports the *P* value as >0.05, <0.05, <0.01, or <0.001. The calculation of the *P* value takes into account the number of comparisons made. If the null hypothesis is true (all data are sampled from populations with identical distributions, so all differences between groups are due to random sampling), then there is a 5% chance that at least one of the posttests will have *P* < 0.05. The 5% chance does not apply to each comparison but rather to the entire family of comparisons.

### 2.4. Piling Activity Details

For the piling of the 56 monopile foundations at the Blighbank, a hammer IHC hydrohammer S1200, operated from the support vessel Svanen, was used. The hammer featured a maximum power of 1200 kJ. The average energy used for each stroke was 706 kJ (Table 2). For the installation of the 49 jacket foundations at the Thortonbank, the piling of 196 pinpiles was required. The hammer used was an IHC hydrohammer S-800 featuring a maximum power of 800 kJ for a nominal power of 720 kJ. Average energy used for each stoke was 412 kJ. The hammer log did not record a time stamp for every blow along with the other information, hampering a direct comparison between the records and the hammer log.

### 2.5. Major Behavioural Disturbance Levels for Marine Harbour Porpoise

Even if underwater noise produced by human activities is known to produce effect to the marine life, including fishes or birds, we propose an illustration to compare our data and model results with known level for the most common marine mammal present in Belgian water. For the harbour porpoise *Phocoena phocoena,* a major behavioural disturbance level, is found above *L*_*p*−*p*_ = 155 dB  re  1 *μ*Pa [13]. 

### 2.6. Regression Model for Noise Propagation

A linear regression model based on the ordinary least square (OLS) was computed from the data presented at Table 1:

(4) Lz−p=−27.4log⁡(d)+270.7 dB  for  monopileLz−p=−27.4log⁡(d)+259,5 dB  for  jacket,

in which *d* is the distance to the source. It has a transmission loss of 27.4log⁡(*d*) ranging within the 95% confidence interval from 30.5 to 24.3log⁡(*d*). That model is further modified by the addition of an absorption term making use of absorption coefficient of 0.0004 dB/m as proposed by [13] and the final model reads

(5) Lz−p=−27.4log⁡(d)+270.7 dB−0.0004d           for  monopileLz−p=−27.4log⁡(d)+259,5 dB−0.0004d            for  jacket.

## 3. Results

### 3.1. Underwater Noise Sound Pressure and Exposure Levels

The highest normalised *L*_*z*−*p*_ of 194 dB re 1 *μ*Pa was observed for the piling of the B10 monopile at the Blighbank, while for the piling of the jacket pinpiles a maximum of 189 dB re 1 *μ*Pa was observed (CG3) at the Thorntonbank (Table 3). The lowest *L*_*z*−*p*_ value of 172 dB re 1 *μ*Pa was observed for the piling of the jacket CB6, while the lowest *L*_*z*−*p*_ for monopiles was 179 dB re 1 *μ*Pa. The piling of the jacket foundation CG3 and the piling of the monopile A02 exert similar normalized *L*_*z*−*p*_ values of about 186 dB re 1 *μ*Pa. Some lower normalized *L*_*z*−*p*_ (by 15 to 20 dB re 1 *μ*Pa) is observed for the piling of the jacket CB6.

Normalized maximum SEL values range between 151 and 178 dB re 1 *μ*Pa^2^s. The maximum observed normalised SEL for jacket foundation piling was 178 dB re 1 *μ*Pa^2^s (CG3), while the maximum observed normalized SEL for monopiles (B10) was some 10 dB lower with a maximum of 166 dB re 1 *μ*Pa^2^s. Normalized mean SELs show similar behaviour with the highest value of 168 dB re 1 *μ*Pa^2^s measured at CG3 and the lowest value for jacket piling of 145 dB re 1 *μ*Pa^2^s (CB6). Normalized mean SELs for both steel monopile are in between with 168 dB re 1 *μ*Pa^2^s for B10 and 164 dB re 1 *μ*Pa^2^s for A02. Whereas statistically significant differences were detected between the four piling events for normalized maximum SEL (Kruskal-Wallis test: *P* = 0.016) and mean SEL (*P* = 0.020), *post hoc* multiple comparisons revealed differences only between the two jacket piling events (*P* = 0.008 and *P* = 0.018, resp.).

### 3.2. Underwater Noise Spectra

For both monopile and jacket piling, the strongest underwater noises were emitted between 60 to 2000 Hz. Moreover, while the shape of the spectra are similar in the frequency domain 100 to 500 Hz, the spectra showed more isolated peaks for the jacket piling than for the monopiling, for which only one larger peak was found. The decay of the spectra showed a similar slope for both foundation types.

On average, a jacket foundation required about three times more blows per foundation (Table 4) than a monopile. When that parameter was normalized to MW installed, 57% more blows/MW installed were needed for jacket foundations than for monopile. Moreover, the average piling time required was higher for a jacket foundation than for a monopile (factor 2.5) and remained somewhat higher when normalized to MW installed (factor 1.3).

### 3.3. Noise Propagation and Attenuation

For both farms, the propagation model (Figure 2) is used to compute the extent of the zone wherein noise levels exceeded the major behavioural disturbance level for harbour porpoises. 

The simple model used is an approximation of the exact situation. That zone of the North Sea features complex geomorphology on a shallow water environment that may induce more complicated propagation and attenuation for underwater sound waves. Nevertheless, when taking into account the variability found on the production of the noise itself (Table 2), the first approximation that is represented by the model is acceptable for the purpose of an estimation of a radius of major behavioural disturbance for marine mammals around a construction place. 

The zone of major behavioural disturbance for harbour porpoises was estimated by the model to a radius of 8 km around the jacket piling location, while that radius extended to 16 km from the monopile piling location.

### 3.4. Cumulative Sound Exposure Level

The mean number of strokes required for the complete piling of one monopile foundation was 3010 strokes (Table 4). As 3010 strokes represent an increase of the normalized @750 m mean SEL of 35 dB (10 log_10_(3010)), the mean cumulative SEL for monopile was estimated at 196 dB re 1 *μ*Pa²s. The mean duration of piling for one foundation was 120 min. A mean number of 9476 strokes were required for the installation of one jacket foundation. This represented an increase of 40 dB, giving a cumulative normalized SEL of 196 dB re 1 *μ*Pa²s @750 m. The mean duration of piling for one jacket was 319 min. The same cumulative SEL values were, hence, observed for both foundation types, but the disturbance time for jacket foundations lasted for longer than that for monopile foundations. 

## 4. Discussion

As expressed earlier, the piling work linked to the installation of the jacket foundation requires the piling of four pinpiles, while the monopile design requires the piling of only one large monopile. Jacket foundations may, however, accommodate larger turbines than monopiles [4]. A less powerful hammer can be used for the installation of the jacket foundations than that for the monopile foundations. However, a jacket design requires longer piling time than the monopile design (mean time of 319 min for jacket against 120 min for monopile), but at lower noise levels with a normalized *L*_*z*−*p*_ of maximum 194 dB re 1 *μ*Pa for a monopile against 189 dB re 1 *μ*Pa for a jacket. The installation of jacket foundations, hence, impacts a smaller zone, but for a longer period of time. 

In terms of energy, the total piling energy needed to achieve the complete construction of the C-Power project, phases 2 and 3 at the Thorntonbank (49 jacket foundations), was just above 0.19 TJ (Table 2), while the same figure for the Belwind wind farm implanted at the Blighbank and featuring 56 monopile foundations was 0.12 TJ. The overall message is that more energy was used and, therefore, transmitted to the environment for the installation of the new C-Power wind farm than that for the installation of the Belwind wind farm. This is further confirmed by the SEL data (Table 3) featuring a maximum value for the normalized SEL of 178 dB re 1 *μ*Pa^2^s for the C-Power project wind farm against 166 dB re 1 *μ*Pa^2^s for the Belwind wind farm.

When underwater noise is generated by pile driving, the size of the pile, power of the pile driver (hammer), and sedimentological and geological properties are important variables, affecting the effective underwater noise produced. For similar sediment properties, using a larger pile driver would generate less noise because of a lower impact velocity applied when hammering [11]. It could also be economically more efficient to use a large pile driver operated at 2/3 of its nominal power than a smaller one used at its maximum power. The use of a less powerful hammer (800 kJ) for pinpiling (versus 1200 kJ for monopiling) in conjunction with the use of smaller pinpiles produced lower *L*_*z*−*p*_ values than those for the monopiling at the Blighbank (some 5 dB re 1 *μ*Pa @750 m). The higher SEL identified for the piling of jacket CG3 (Table 2) in comparison with the piling of the jacket CB6 is most probably related to the use of the hammer at a higher power, even if we cannot demonstrate that relation due to the unavailability of a timestamp for every blow. However, to conclude the differences observed between pinpiling and monopiling, a significant difference was found within the pinpiling group (Table 3). This significant difference can be explained by the fact that the piling of one of the jackets (CB6) required only a third of the mean energy used for the installation of the other jackets (Table 2). This could indeed be related to the small scale local differences in sedimentological and geological properties.

Nevertheless, when renormalizing these data to the installed power, the message is different with a little lower average energy per MW used for the jacket foundation (665 MJ/MW) than that for the monopile foundation (721 MJ/MW). While jacket piling used less piling energy per MW, the average duration of piling per installed MW remained 26% higher with 55 minutes for a jacket and only 41 minutes for a monopile. However, an even better normalization would be obtained when standardising to the MW produced instead of the MW installed. Such standardisation would, however, be premature at this moment, since the wind farms are either operational for a short period of time (Belwind) or not yet operational at all (C-Power, phases 2 and 3). 

For both monopiling and jacket installed in the BPNS, cumulative SEL of 196 dB re 1 *μ*Pa²s @750 m was found. Comparison with the available data for the Q7 wind farm [10] located in Dutch waters and featuring 4 m diameter monopiles was possible after a renormalization at 750 m. Some 13 dB higher cumulative SEL was computed (209 dB re 1 *μ*Pa²s). Unfortunately, other comparisons based on that variable are difficult to make since primary data are missing. Adapted from [11], zero to peak levels ranging between 185 and 199 dB re 1 *μ*Pa for a pile diameter ranging between 3,3 and 4,7 m were observed in various wind farms located in German and UK waters. These results are of the same order of magnitude and coherent with what was observed in the BPNS wind farms.

**Figure 1:**
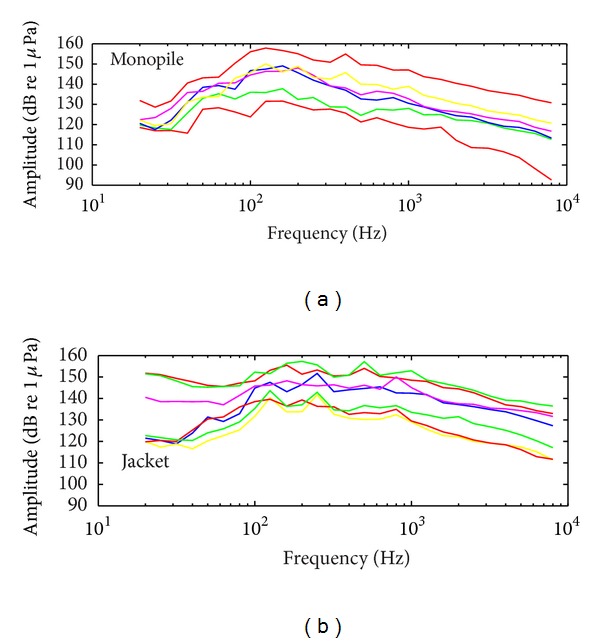
1/3 octave spectra of the underwater noise of the Blighbank monopiling and the C-Power jacket piling.

**Figure 2:**
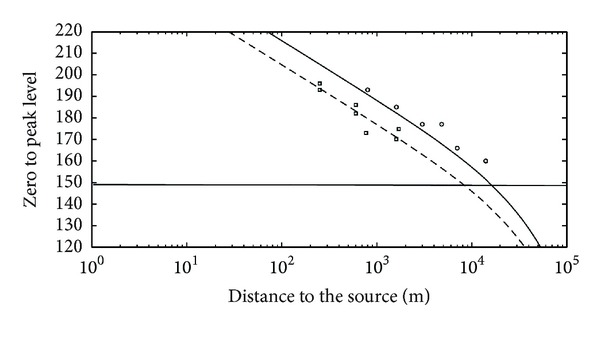
Application of the propagation model to jacket piling (dashed line) and monopiling (plain line). Squares and circles are the measured *L*_*z*−*p*_, respectively, for jacket and monopile (Table 1) while the horizontal line at 149 dB re 1 *μ*Pa represents the level (*L*_*z*−*p*_) for major behavioural disturbance for harbour porpoise [13].

Some of the levels observed here for both the monopile or jacket type foundations installation exceed the 185 dB re 1 *μ*Pa permitted by the Belgian MSFD descriptor 11. This indicates that future offshore wind farms will need to take mitigating measures during construction. Different methods exist [11, 14]. One of these is the air bubble curtain method [15] that could reduce the levels (both *L*_*z*−*p*_ and SEL) by about 14 dB. These values were obtained inside a port and such technique remains to be validated at sea, with, for example, strong tidal current. A current of 1 m/s, which is not uncommon for the BPNS, may indeed induce a drift of the bubble curtain of about 70 m for a bottom depth of 20 m [11]. New difficulties may arise when the sleeve may be in contact with the pile due to the tidal current. For bubble curtains, size of the bubble has an impact on sound insulation [14, 15]. A second method often preferred by the industry for sound isolation is the use of pile sleeves made from various material including foam or air [11, 14]. This last method can achieve a sound reduction of 20 to 25 dB for low frequencies where the maximum noise is produced (Figure 1). These methods, if they were used in conjunction with piling works, would have reduced the produced noise to levels below the Belgian MSFD requirements.

## 5. Conclusion

- While jacket foundations involved smaller diameter pinpiles and while the emitted noise levels normalized at 750 m *L*_*z*−*p*_ values are lower than those for monopiling, therefore impacting a smaller zone, the overall energy needed for the complete piling was 58% higher for the 49 jackets than for the 56 monopiles. The normalized @750 SEL was also higher for jacket than for monopile foundation piling.
- When normalized to installed MW the figure is inversed and average energy needed by installed MW is 8% lower for jacket than for monopile.
- Finally, for both maximum and mean normalized @750 m SEL, no statistically significant difference on the emitted underwater noise between pinpiling and monopiling could, however, be observed.
- The radius for major behavioural disturbance was modelled to reach 16 km for monopile and 8 km for jacket.
- Some measurements are above the Belgian MSFD requirements and those for monopile as well as for jacket. Use of mitigation measures could have reduced the produced noise below these requirements.

## Figures and Tables

**Table 1:** Geographic position, peak level (*L*_*z*–*p*_), and distance from the piling location of the underwater noise measurements at the Blighbank site (monopiles A02 and B10) and at the Thorntonbank site (jackets CG3 and CB6).

Position start recording (WGS84)		Peak level (dB) *L*_*z*–*p*_	Distance (m) from piling location
Latitude	Longitude		
Monopile A02			
51°40.39′	2°50.03′	177	~3000
51°39.41′	2°50.64′	177	~4820
51°38.25′	2°51.25′	166	~6990
Monopile B10			
51°34.59′	2°57.31′	159	~14150
51°38.52′	2°48.16′	185	~1580
51°38.50′	2°47.44′	193	~770
Jacket CG3			
51°33.92′	2°58.94′	192	~250
51°51.34′	2°58.36′	187	~500
51°33.96′	2°58.93′	196	~250
Jacket CB6			
51°33.07′	2°53.94′	182	~600
51°32.96′	2°52.59′	175	~1700
51°32.65′	2°53.42′	172	~750
51°32.22′	2°53.01′	171	~1600

**Table 2:** Summary statistics of the piling activities of monopile A02 and B10 and jacket foundations CB6 and CG3, targeted in this study, as well as the averages and total (where appropriate) for the 56 monopiles installed at the Blighbank (source: Belwind) and the 49 jacket installed on the Thorntonbank (source: C-Power).

	Monopile piling activities (pile diameter = 5 m)					Jacket piling activities (pinepile diameter = 1.8 m)				
	Unit	A02	B10	Average	Total	Unit	G3	B6	Average	Total
Pile length	m	55	63	54		m	48	21	37	—
Mass	t	401	453	375		t	96	46	77	—
Number of strokes required		2114	3848	2982	168550		13321	4288	9476	464328
Average energy per stroke	kJ	642	839	706		kJ	436	321	412	
Duration of piling	min	64	163	120	6779	min	405	162	319	15646
Net piling frequency	Number of strokes/minute	42	39	40		Number of strokes/minute	About 40	About 40		
Total energy	MJ	1356	3224	2084	118909	MJ	5805	1376	3909	191531

**Table 3:** Normalized @ 750 m zero to peak sound pressure level (*L*_*z*–*p*_) in dB re 1 *μ*Pa. Normalized @ 750 m mean and maximum sound exposure levels (SEL) in dB re 1 *μ*Pa^2^s.

	Record	Norm. *L*_*z*–*p*_ @ 750 m	Norm. mean SEL @ 750 m	Norm. max. SEL @ 750 m
Monopile A02	1	186	161	164
	2	189	164	166
	3	180	160	164

MonopileB10	1	194	162	166
	2	190	168	162
	3	179	163	166

Jacket CG3	1	185	168	174
	2	189	168	178
	3	186	168	175

Jacket CB6	1	180	155	159
	2	172	145	151
	3	176	150	152
	4	180	152	157

**Table 4:** Characterization of the monopile and jacket piling activities. Normalized maximum sound exposure level (norm. max. SEL @ 750 m).

Foundation type	Monopile (3 MW)	Jacket (6 MW)
Average no. of blow/foundation	3010	9476
Average no. of blow/MW installed	1021	1612
Average energy (MJ)/blow	0.7	0.4
Average energy (MJ)/foundation	2123	3909
Average energy (MJ)/MW installed	721	665
Norm. max. SEL @750 m (dB re 1 *μ*Pa²s)	166	178
Average duration of piling (min)/foundation	120	319
Average duration of piling (min)/MW installed	41	55
Average piling frequency (blow/min)	25	30
